# Supplementary figures and images for: A data-driven approach for real-time soft tissue deformation prediction using nonlinear presurgical simulations
Source: PLoS One. 2025 Apr 14;20(4):e0319196. doi: 10.1371/journal.pone.0319196 (PMC11996222; doi:10.1371/journal.pone.0319196)

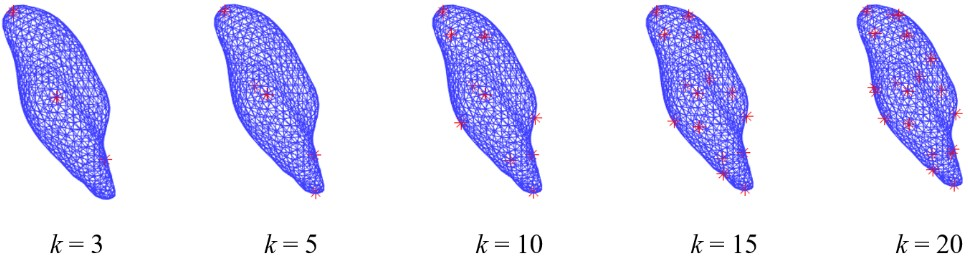

Supplement: S1 Fig — Starting at the initial node index of 97, the red asterisks represent the centers obtained by the revised K-center clustering algorithm. k = 5 is eventually selected for implementations in this paper. (TIF) [file pone.0319196.s001.tif]
