# Supplementary material for: A data-driven approach for real-time soft tissue deformation prediction using nonlinear presurgical simulations
Source: PLoS One. 2025 Apr 14;20(4):e0319196. doi: 10.1371/journal.pone.0319196 (PMC11996222; doi:10.1371/journal.pone.0319196)
Supplement: Attachment — Submitted filename: REVIEW.pdf [file pone.0319196.s002.pdf]

Summary of the work:

In this work, the authors present a ML surrogate model for FEM models for predicting tissue deformation given the displacements of some points (Fiducial Markers (FMs)). These FM are introduced during surgery procedures for real-time tracking of the region of interest (i.e., a tumor, a organ) through MRI or CT imaging, to then use then to track displacement with a lower fidelity method. With that patient-specific pretrained model, then a NN receives the displacements of the FM and transforms it to the latent space so the use the decoder of the VA to obtain the deformation of the region of interest.

Overall, I see the potential of the work and its application to clinical practice as it would help for more precise surgical interventions as it would allow a real-time representation of the region of interest with lower fidelity systems such as ultrasound imaging. The proposed data-driven approach makes sense as it allows taking advantage of the information from the FMs to perform real-time simulations and, in a further step, allow generalization across patients. However, I think there are several important concerns that should be addressed for publication, especially regarding the novelty of the work, the ML model, the information provided by the figures, and the flow of the paper.

Reviews

1. My first concern is regarding the novelty of the work. At lines 45-47 the authors state that existing literature rarely focuses on ML+FEM approaches. However, there are several works about combining FEM with ML to perform real-time deformation simulations. Just by doing a quick search I found:

Related works to the paper:

https://www.sciencedirect.com/science/article/pii/S0933365716304687

https://www.sciencedirect.com/science/article/pii/S0010482517303177

https://www.sciencedirect.com/science/article/pii/S0952197623013349

https://www.sciencedirect.com/science/article/pii/S0895611122001355

A review:

https://aapm.onlinelibrary.wiley.com/doi/full/10.1002/mp.14602

Additionally, some works that already presented FEM+ML to calculate organ deformations: https://www.sciencedirect.com/science/article/pii/S0957417416306728

I would suggest that the authors should clarify in the introduction what they contribute compared to these works that employ similar approaches. In the same way that they review some works of FEM approaches (line 35), they should also mention the FEM+ML approaches and the benefits of the proposed work versus literature work.

2. I think that figure 1 is key to fully understand the procedure and needs improvement, especially regarding the flow of the figure and how the data is employed. As it is now, it's a bit chaotic and not clear. Maybe it would benefit from dividing the figure with a. b. c… subfigures to make it clearer. In this way, the caption can explain in more details each part of the figure and then I think it may support the explanation in the manuscript in the material and methods section, as the text could reference each part of the figure as the manuscript explains the "creation of deformation…", "encoding of deformation vectors", "deformation prediction with NN". Also, in the figures the *during the surgery* and *before the surgery* texts are not clear to where they span.

3. The authors show the methodology using head-and-neck (H&N) tumor and reference Figure 2, but in Figure 2 they also show other geometric models that are not commented. I recommend that they present those geometries and then establish that they will focus on H&N for explanation purposes, although I doubt about the value added by that figure.

4. In Figure 3 they show the force fields. Scale bar values are not clear. For each subfigure, n=1, n=3, and so on, what are the differences between those 3 geometries?

5. It does make sense to build an autoencoder to reduce the dimensionality of the problem and ease the deformation prediction. However, I have some doubts about the autoencoder model and training, there are few details about this. How is it trained? Is it the same dataset Gtrain, Gvalid, Gtest that is used to train the NN? In that case, wouldn't the NN replicate the same latent space for which the AE was trained?

6. Line 163-165 parametric studies for the NN. However, could not find how they decided the optimal NN architecture of [n1, n2, n3] = [1024, 512, 256]. The parametric studies section is rather scarce, it does give little information.

7. I think it presenting the results and then presenting the parametric studies, number of FMs, and latent space does not help to the flow of the paper. I believe the paper would benefit from rearranging and reorganizing.

Minor things:

Reference 5, last accessed missing

Line 265 extra space in H& N

Conclusions:

I believe that the work needs some improvements to finally consider it for publication.
