# Supplementary material for: A data-driven approach for real-time soft tissue deformation prediction using nonlinear presurgical simulations
Source: PLoS One. 2025 Apr 14;20(4):e0319196. doi: 10.1371/journal.pone.0319196 (PMC11996222; doi:10.1371/journal.pone.0319196)
Supplement: Attachment — Submitted filename: Response to Reviewers.pdf [file pone.0319196.s003.pdf]

Revision overview: We thank both reviewers for their thoughtful comments and suggestions. We have replied to each comment below, and marked changes to the manuscript in red.

Reviewer #1: This paper presents a methodology to predict in real-time the deformation of human tissues during image-guided surgeries. A dataset of FEM simulations under a range of force conditions is created with geometric models of a patient's region of interest during surgery. The training dataset is employed to train an autoencoder, which develops a latent space representation capable of encoding tissue shape deformations. Subsequently, a neural network and a regression model are trained to link specific significant displacements to these latent space vectors of the deformation fields. The predicted latent space vectors are then decoded to obtain the deformation field vectors corresponding to the deformed tissue and predict in real time the deformed tissue. While the paper is globally well written and clear, there are some open issues which should be addressed before publication.

1. *Even if you choose non-linear material properties, the equation used for the FEM problem is for a linear problem. How do you explain this?*

   A: In our FEM simulation, the load on the external soft tissue surface, modeled as a force field boundary condition, was applied incrementally with a time history. The linear equation F=kx is simply a linear solver with the Newton-Raphson algorithm for one "incremental step" (to not be confused with numerical "solving steps", we call it one "increment" hereafter). This means that despite simulating one increment linearly, the overall simulation history is considered nonlinear -- the initial deformed configuration and nodal variables of each increment are taken from the end frame of the previous simulation increment. Combined with material nonlinearity, we believe this simulates nonlinear behaviors of soft tissue deformation.

   We have made the following change at Line 130 in the manuscript to better explain the nonlinearity of our simulation pipeline: "In essence, the nonlinearity of our simulation arises from both nonlinear material properties and nonlinear simulation schemes. This is sufficient to capture most of the nonlinear deformation behaviors of the target soft tissues. ".

2. *Why do you use the same mechanical properties for the simulation of the three tissues? Do you have a reference to justify the parameters and all the properties in Table 1?*

   A: Thanks for the reviewer's suggestion. Our main goal is to show the efficacy of our framework regarding predicting full-field deformation given fiducial markers' displacements. To make the corresponding studies comparable in terms of overall displacement magnitudes, we kept the materials properties the same across the

different examples we present. Since we generated latent distribution based on material-property-agnostic deformation data, we believe that the machine learning model would yield equally accurate results with whatever material properties we choose as long as the soft tissue deformation magnitude falls within a similar range.

However, we do acknowledge the necessity to demonstrate the aforementioned point, therefore we identified from the literature the material properties of kidney and aorta tissue and changed them accordingly in the simulation model. We find that the kidney has been modeled as a neo-Hookean solid with an elastic modulus of 180 kPa, while the aorta tissue has been modeled as a neo-Hookean solid with an elastic modulus of 250 kPa. Note that we too model these two tissues as neo-Hookean solids.

Table 1. Simulation results of average nodal deformation (mm) of kidney and aorta soft tissue models with newly found realistic material properties.

	**Average nodal deformation (mm)**				
**Example No.**	1	2	3	4	5
**Kidney model with new properties**	11.35	24.18	6.35	3.77	7.72
**Aorta model with new properties**	3.39	4.27	5.49	1.98	4.16

We performed batch simulations for the kidney (in a total of 20 simulations) and aorta (in a total of 30 simulations) models with the newly found material properties from literature and similar boundary conditions, and reported 5 of each model in the above table. We found that after performing the same simulation procedures, the average of the average nodal deformation for the kidney and aorta is 12.46 mm and 4.73 mm, respectively. This falls within the range of what we have simulated with the material properties of the head and neck tumor, therefore we believe this is sufficient to demonstrate that our data-driven framework will work well with these two new material properties and similar load conditions.

We have made the following change at Line 97 in the manuscript to better explain the big picture of our idea and resolve this issue: “It is worth noting that we used the same material properties from the H&N tumor for our kidney and aorta aneurysm models as well, since the major aim of this research is to show the capability of our data-driven pipeline on full-field deformation prediction given only a few displacements values of FMs, rather than generating realistic deformation fields for each respective model. We hypothesize that our trained machine learning model would yield equally accurate results with whatever material

properties we choose as long as the soft tissue deformation magnitude falls within a similar range. ”.

We list the reference for the aforementioned kidney and aorta tissue material properties below:

Aorta tissue: Okamoto, Ruth J., et al. "Mechanical properties of dilated human ascending aorta." *Annals of biomedical engineering* 30 (2002): 624-635.

Kidney: Karimi, A., and A. Shojaei. "Measurement of the mechanical properties of the human kidney." *Irbm* 38.5 (2017): 292-297.

3. *Line 92: “Deformations are primarily driven by the forces generated in the tissue’s interstitial environment”. Can you explain better the nature of these forces? Why are they generated? How? Where? Why do they influence tissue deformation? Do you have some references for these forces?*

   A: We generated the force fields by (a) randomly creating up to three nodal forces on the surface of the tissue, and (b) applying the Laplace-Beltrami operator to smooth the aforementioned nodal forces to the entire tissue surface. The purpose of force field generation on the tissue surface is to create smooth yet non-homogeneous distributed loads (force fields) that mimic a realistic environment a tissue is embedded in, where the surface contact forces applied to the tissue could potentially be from arbitrary directions with magnitudes causing mild deformation. To make sure we create a dataset with a large variety of force fields based on the above conditions, we randomly pick up to three surface nodes and sample their magnitudes in either a uniform or a Gaussian way.

   The force field smoothing has been re-depicted with a newly created Figure 3. And we have made the following change in Line 138 of the manuscript to explain our force field generation better: “As shown in Figure. 4, we first generate a few nodal forces on the surface of the model by randomly selecting positions, magnitudes, and directions of the loads; then we perform spectral decomposition of the discretized Laplacian of the tumor's surface mesh to obtain a discretized Laplacian-Beltrami operator (LBO) that can smoothly spread the nodal forces into smoother force field distributions around them.”.

4. *If Kin and Kout represent matrices they should be written in bold.*

   A: We thank the reviewer for the good catch! We have made Kin and Kout bold to indicate that they are matrices. The corrected sentence is now red in LIne 112.

5. *Regarding the creation of the training and test datasets: Is the difference in the generated force field only in the magnitude and direction of the forces? Is the application point of the force always the same? If so, how do you choose the application point? The caption in figure 3 states: “randomly assigned node on the outer surface as the loading position,” but this is not explained in the main text.*

A: As we mentioned in the previous question, we randomly picked the load positions, directions, and magnitudes to try to create a diverse enough deformation dataset. We also want to clarify that as we apply our system, we do not need to input the location of the realistic load -- the only input should be the geometry of the model, the location of fiducial markers, and fiducial marker displacements. As such, our data-driven framework is load-condition-agnostic and would perform better if the training dataset had richer selections of loads.

6. *What is the computational time of the FEM simulations?*

   A: We thank the reviewer for the question. The simulation time of our FEM is on average 5 mins, given specific boundary conditions on the surface of the tissue model. In a real intraoperative application, however, it could be challenging to obtain and keep tracking accurate load conditions on the outer surface of the tissue. Therefore, the actual time could be much longer given that more time is needed to accurately model the boundary conditions. We have added the following sentence in the Conclusion section of the manuscript to highlight the computational time advantage of our framework: "After the NN is trained, the deformation prediction of one case takes less than 0.5 s, compared to the computational time of around 5 mins for each simulation via the FEM method. This demonstrates the proposed approach's capability in real-time tracking of intraoperative soft tissue deformations with high reconstruction fidelity.".

7. *Can you give more information about the variability of the dataset in the displacement field for each of the three cases tested?*

   A: The following table shows the variability of our simulation results used to generate our training and testing dataset.

	**H&N tumor**	**Kidney**	**Aorta**
**Max. nodal deformation (mm)**	18.33	25.72	15.74
**Min. nodal deformation (mm)**	0.79	0.45	0.34
**The standard deviation of nodal deformation (mm)**	3.87	6.66	2.14

Is the variability in the range of clinically relevant data?

A: Yes, the variability in our dataset is within the range of clinically relevant data. While it is challenging to find exact reference values for each specific geometry, several papers cite the range of nodal deformations during image guided liver

surgery as between 5 to 20mm (Clements et al. 2011 & 2017), as well as aforementioned deformation modeling papers which simulated displacements up to 15mm (Lorente et al. 2017).

Clements, Logan W., et al. "Deformation correction for image guided liver surgery: An intraoperative fidelity assessment." *Surgery* 162.3 (2017): 537-547.

Clements, Logan W., et al. "Organ surface deformation measurement and analysis in open hepatic surgery: method and preliminary results from 12 clinical cases." *IEEE transactions on biomedical engineering* 58.8 (2011): 2280-2289.

Lorente, Delia, et al. "A framework for modelling the biomechanical behaviour of the human liver during breathing in real time using machine learning." *Expert Systems with Applications* 71 (2017): 342-357.

8. *You should provide more results for each example about the learning process of the NN. What is the behavior of the validation loss function with respect to the training loss function?*

   A: We monitor the loss curves during training to ensure there is not an increase in the validation curve while the training curve continues decreasing - this would indicate overfitting to the training data. We briefly address this point in Sec. Deformation Prediction with NN, but feel it does not warrant new figures as this concept is well established within the field of machine learning and may be either superfluous information or potentially confusing information depending on the reader's background.

   Why do you use the same neural network architecture for the three examples?

   A: Good question - we tested out many different hidden layer structures and found empirically that the [1024, 512, 256] structure appropriately balanced generalizability without a strong propensity to overfit to training data across all models. Rather than fine-tuning the model to obtain marginally better results for each geometry, we present a single architecture that performs well across all patient-specific geometries. We have added several sentences to clarify this point in Sec. Deformation Prediction with NN.

9. *In the limitations, you state: "If the initial geometry of the sample is changed, the NN may fail to reconstruct its deformation." Can you test the influence of the initial geometry on the results of your model? What is the clinical applicability of the model if we need to train another NN for each new geometry?*

   A: Thank you for the question - neural networks typically struggle with out-of-bounds prediction, meaning that a model trained on deformations for a specific geometry will struggle to generate quality predictions on a new, unseen geometry. For example - this would be like taking a model trained on patient A's H&N tumor, and then using that trained model to guide the surgery on patient B's tumor. It is much more clinically appropriate to train a new model based on patient

specific geometries and deformation to ensure the highest degree of accuracy when using the model to guide a surgery. That said, we train the models on a diverse range of deformations for a specific geometry to ensure the model can accurately predict a broad range of deformations that might be seen during surgery.

Reviewer #2: Summary of the work:
In this work, the authors present a ML surrogate model for FEM models for predicting tissue deformation given the displacements of some points (Fiducial Markers (FMs)). These FM are introduced during surgery procedures for real-time tracking of the region of interest (i.e., a tumor, a organ) through MRI or CT imaging, to then use then to track displacement with a lower fidelity method. With that patient-specific pretrained model, then a NN receives the displacements of the FM and transforms it to the latent space so the use the decoder of the VA to obtain the deformation of the region of interest. Overall, I see the potential of the work and its application to clinical practice as it would help for more precise surgical interventions as it would allow a real-time representation of the region of interest with lower fidelity systems such as ultrasound imaging. The proposed data-driven approach makes sense as it allows taking advantage of the information from the FMs to perform real-time simulations and, in a further step, allow generalization across patients. However, I think there are several important concerns that should be addressed for publication, especially regarding the novelty of the work, the ML model, the information provided by the figures, and the flow of the paper.

1. *My first concern is regarding the novelty of the work. At lines 45-47 the authors state that existing literature rarely focuses on ML+FEM approaches. However, there are several works about combining FEM with ML to perform real-time deformation simulations. Just by doing a quick search I found:*
   Related works to the paper:
   https://www.sciencedirect.com/science/article/pii/S0933365716304687
   https://www.sciencedirect.com/science/article/pii/S0010482517303177
   https://www.sciencedirect.com/science/article/pii/S0952197623013349
   https://www.sciencedirect.com/science/article/pii/S0895611122001355
   A review:
   https://aapm.onlinelibrary.wiley.com/doi/full/10.1002/mp.14602
   Additionally, some works that already presented FEM+ML to calculate organ deformations:
   https://www.sciencedirect.com/science/article/pii/S0957417416306728
   I would suggest that the authors should clarify in the introduction what they contribute compared to these works that employ similar approaches. In the same way that they review some works of FEM approaches (line 35), they should also mention the FEM+ML approaches and the benefits of the proposed work versus literature work.

   A: Great point, thank you for pointing us towards all of these prior works. We feel that our paper is still well situated within the space, and have added a paragraph discussing these works, and make the case that our fiducial marker based approach makes use of an already common method for registering intraoperative images, and extends this to an ML based deformation pipeline.

2. *I think that figure 1 is key to fully understand the procedure and needs improvement, especially regarding the flow of the figure and how the data is*

employed. As it is now, it's a bit chaotic and not clear. Maybe it would benefit from dividing the figure with a. b. c… subfigures to make it clearer. In this way, the caption can explain in more details each part of the figure and then I think it may support the explanation in the manuscript in the material and methods section, as the text could reference each part of the figure as the manuscript explains the "creation of deformation…", "encoding of deformation vectors", "deformation prediction with NN". Also, in the figures the during the surgery and before the surgery texts are not clear to where they span.

A: Great point, we agree that Figure 1 was rather dense and difficult to understand. We have broken down figure 1 into 4 subfigures which more clearly distinguishes the different aspects of training and deployment, and more closely aligns with the respective sections.

3. *The authors show the methodology using head-and-neck (H&N) tumor and reference Figure 2, but in Figure 2 they also show other geometric models that are not commented. I recommend that they present those geometries and then establish that they will focus on H&N for explanation purposes, although I doubt about the value added by that figure.*

   A: Good point. We feel it is important to have visualizations of the default geometries used throughout the paper, and have added a sentence at the end of the material and methods section to clarify that the kidney and aorta aneurysm geometries are presented but will be used later in the paper.

4. *In Figure 3 they show the force fields. Scale bar values are not clear. For each subfigure, n=1, n=3, and so on, what are the differences between those 3 geometries?*

   A: We thank the reviewer for this great catch! We have recreated Figure 3 in the manuscript with a much clearer depiction and larger color bars. The difference between the three subfigures is supposed to show the discrepancy of load magnitudes along with three directions (i.e., XYZ).

5. *It does make sense to build an autoencoder to reduce the dimensionality of the problem and ease the deformation prediction. However, I have some doubts about the autoencoder model and training, there are few details about this. How is it trained? Is it the same dataset Gtrain, Gvalid, Gtest that is used to train the NN? In that case, wouldn't the NN replicate the same latent space for which the AE was trained?*

   A: *Gtrain, Gvalid, and Gtest are different deformation datasets without any duplications and overlaps. We believe that our created dataset (or in a realistic application, one should create a deformation dataset by randomly sampling force fields that) is diverse enough to include almost all possible deforming cases given different fiducial marker displacements. Under such consideration, the latent*

distribution our AE created will be close to the "real" distributions of deformation cases, i.e., the latent space can capture the characteristics of deformation fields with different fiducial marker displacement patterns. To validate this hypothesis, we also generate Gtrain, Gvalid, and Gtest differently -- by sampling the loads from different probability distributions and making sure the sub-distributions of Gtrain, Gvalid, and Gtest are not equal.

6. *Line 163-165 parametric studies for the NN. However, could not find how they decided the optimal NN architecture of [n1, n2, n3] = [1024, 512, 256]. The parametric studies section is rather scarce, it does give little information.*

   A: Great point, thank you. We do not perform a rigorous parametric study to determine the optimal hidden layer structure, and have removed this line so we do not give the reader that impression. Instead we clarify that we empirically arrived at this structure. In developing the models we tested out many different hidden layer structures and found empirically that the [1024, 512, 256] structure appropriately balanced generalizability without a strong propensity to overfit to training data across all models. Rather than fine tuning the model to obtain marginally better results for each geometry, we present a single architecture which performs well across all patient specific geometries. That said, we do perform parametric studies to determine the input/output layer dimensions of the NN, which correspond to the number of FMs and the latent dimension. We have added several sentences to clarify this point in Sec. Deformation Prediction with NN.

7. *I think it presenting the results and then presenting the parametric studies, number of FMs, and latent space does not help to the flow of the paper. I believe the paper would benefit from rearranging and reorganizing.*

   A: While we agree that there is an element of selecting the number of FMs and latent space dimension that is related to the methods, we feel that the discussion presented as to the reasoning and implications of these selections is best suited to appear with the results/discussion section. Overall, the methodology presented in the paper does not change based on the number of FMs and latent space dimension, but there are considerations to be taken into account in terms of surgical/clinical feasibility with regards to the number of FMs, as well as real time computation speed and accuracy with regards to the latent space dimension.

8. *Minor things:*
   a. *Reference 5, last accessed missing*

      A: Fixed

   b. *Line 265 extra space in H& N*

      A: Fixed
