# Supplementary material for: A data-driven approach for real-time soft tissue deformation prediction using nonlinear presurgical simulations
Source: PLoS One. 2025 Apr 14;20(4):e0319196. doi: 10.1371/journal.pone.0319196 (PMC11996222; doi:10.1371/journal.pone.0319196)
Supplement: Attachment — Submitted filename: Soft Tissue Revision 2 Response to Reviewers.pdf [file pone.0319196.s004.pdf]

Revision overview: Thank you to both of the reviewers for their insightful comments and questions. We have replied to each point below and marked changes to the manuscript in red text.

Reviewer #1: The authors have adequately addressed the suggestions made by the reviewers, modifying the original manuscript.

1.) *Minor Comment: In equations 1 and 2, the terms corresponding to matrices and vectors should be in bold.*

    A: Thank you for pointing this out, we have bolded the matrices and vectors in these equations.

Reviewer #3: This paper proposes a data-driven method for real-time prediction of soft tissue deformation, aiming to address the shape mismatch between high-resolution preoperative images and low-resolution intraoperative images. The study utilizes the finite element method (FEM) to generate an offline deformation dataset, compresses the high-dimensional deformation field into a low-dimensional latent space via an autoencoder, and trains a neural network (NN) to map the displacement of landmarks to the latent space representation. Finally, the 3D deformation shape is reconstructed through a decoder. The method was tested on three geometric models—head and neck tumors, kidneys, and aortic aneurysms—demonstrating sub-millimeter prediction accuracy and real-time computational performance. However, several issues are noted:

1.) *How is the reliability of the dataset established? Variations in boundary conditions and mesh resolutions in FEM simulations can have a significant impact on the results. However, the paper does not provide evidence to confirm that the results are independent of these factors.*

    A: For force field boundary conditions, we generated them by (a) randomly creating up to three nodal forces on the surface of the tissue, and (b) applying the Laplace-Beltrami operator to smooth the aforementioned nodal forces to the entire tissue surface. The purpose of force field generation on the tissue surface is to create smooth yet non-homogeneous distributed loads (force fields) that mimic a realistic environment a tissue is embedded in, where the surface contact forces applied to the tissue could potentially be from arbitrary directions with magnitudes causing mild deformation. To make sure we create a dataset with a large variety of force field boundary conditions based on the above conditions, we randomly pick up to three surface nodes and sample their magnitudes in either a uniform or a Gaussian way. The force fields are then smoothed using the Laplace-Beltrami operator, the process of which is depicted in Figure 3.

    Since all of our simulations converged successfully, we believe that mesh resolution would not change the results drastically. Therefore, it is not necessary to change mesh resolutions and it won't change the essential conclusion of our

paper.

2.) *Are the references cited in 10–22 really necessary? This section seems to have an unusually large number of citations, are they all essential to support the argument?*

A: We have removed the citations which are not directly related to soft tissue deformation prediction, namely citations [11] Lowe et al. and [22] Jaradat et al.

3.) *The paper is divided into too many small sections, which affects the overall readability. It might be more effective to group the content into three main parts: background introduction, method development, and result discussion, with clear summary paragraphs for each section to enhance clarity.*

A: Thank you for the suggestion. The paper is already primarily organized with the main sections that you suggest; introduction, methods, results/discussion. We further break these sections into subsections and subsubsections which are titled in a smaller font. We feel this structure offers better clarity to the reader rather than simply having a large wall of text under each section with the only distinction being paragraph breaks.

4.) *Why do the head and neck, kidney, and aortic aneurysm models all share the same Young's modulus? The deformation dataset appears to rely on fixed material properties, but there is no validation of how variations in material properties, such as changes in elastic modulus, might influence the results.*

A: Our main goal is to show the efficacy of our framework regarding predicting full-field deformation given fiducial markers' displacements. To make the corresponding studies comparable in terms of overall displacement magnitudes, we kept the material properties the same across the different examples we present. Since we generated latent distribution based on material-property-agnostic deformation data, we believe that the machine learning model would yield equally accurate results with whatever material properties we choose as long as the soft tissue deformation magnitude falls within a similar range.

However, we do acknowledge the necessity to demonstrate the aforementioned point, therefore we identified from the literature the material properties of kidney and aorta tissue and changed them accordingly in the simulation model. We find that the kidney has been modeled as a neo-Hookean solid with an elastic modulus of 180 kPa, while the aorta tissue has been modeled as a neo-Hookean solid with an elastic modulus of 250 kPa. Note that we too model these two tissues as neo-Hookean solids.

Table 1. Simulation results of average nodal deformation (mm) of kidney and aorta soft tissue models with newly found realistic material properties.

	**Average nodal deformation (mm)**				
**Example No.**	1	2	3	4	5
**Kidney model with new properties**	11.35	24.18	6.35	3.77	7.72
**Aorta model with new properties**	3.39	4.27	5.49	1.98	4.16

We performed batch simulations for the kidney (in a total of 20 simulations) and aorta (in a total of 30 simulations) models with the newly found material properties from literature and similar boundary conditions, and reported 5 of each model in the above table. We found that after performing the same simulation procedures, the average of the average nodal deformation for the kidney and aorta is 12.46 mm and 4.73 mm, respectively. This falls within the range of what we have simulated with the material properties of the head and neck tumor, therefore we believe this is sufficient to demonstrate that our data-driven framework will work well with these two new material properties and similar load conditions.

In the “Materials and Methods” section of the paper, we explain this point as follows: “It is worth noting that we used the same material properties from the H&N tumor for our kidney and aorta aneurysm models as well, since the major aim of this research is to show the capability of our data-driven pipeline on full-field deformation prediction given only a few displacements values of FMs, rather than generating realistic deformation fields for each respective model. We hypothesize that our trained machine learning model would yield equally accurate results with whatever material properties we choose as long as the soft tissue deformation magnitude falls within a similar range. ”.

We list the reference for the aforementioned kidney and aorta tissue material properties below:

Aorta tissue: Okamoto, Ruth J., et al. "Mechanical properties of dilated human ascending aorta." *Annals of biomedical engineering* 30 (2002): 624-635.

Kidney: Karimi, A., and A. Shojaei. "Measurement of the mechanical properties of the human kidney." *Irbm* 38.5 (2017): 292-297.

5.) *How were the parameters for the NN’s hidden layers determined? The paper does not provide sufficient explanation or a clear rationale for the choice of the NN’s loss function.*

A: For the NN we used, we tried to make the fully connected structure as simple as possible since it mainly works on mapping between two vectors. We performed test runs over a few three-layer multilayer perceptrons with different numbers of hidden layer neurons, and eventually fixed the structure to the current configuration. For the loss function, we chose to use the mean square error loss (MSELoss) since it is one of the most widely used ones and fits the task we designed in this research.

6.) *Would this method need to be retrained for patients with different constitutions or tissue characteristics? This aspect has not been addressed in the paper.*

A: Yes, the model should be patient-specific (as stated in the last paragraph of the Introduction section) and needs to be retrained for different patients or different tissue properties. However, if it can be experimentally determined that the material properties and shape/structure of tissues (e.g., livers) of two patients are almost identical, the same model could be used to directly guide the intraoperative deformation tracking during surgeries. However, we believe that the main selling point of our paper is to show the capability and efficacy of our proposed framework - a framework that leverages machine learning techniques and fiducial marker displacements to reconstruct the full-field deformation of the tissue. This framework allows surgeons to simulate & train models with given patient-specific properties and parameters before the surgery, which is easy & cost-effective to achieve and can bring benefits to surgery monitoring.

7.) *When compressing the deformation field into a low-dimensional latent space, is there a risk of losing important deformation information? The paper does not discuss this potential concern.*

A: Good question, we address this point in the Parametric Studies: Latent Dimension section. We perform a parametric sweep of latent dimensions and select the dimension according to the “elbow method,” where higher dimensions offer diminishing returns in nodal reconstruction error. We further discuss how the selection of the latent dimension also must consider the added computational cost as well as the interaction with the neural network which maps between the fiducial marker displacements and the latent vector.

8.) *Are the test data generated in the study comparable in complexity to real intraoperative data?*

A: Yes, the test data is comparable in complexity to real intraoperative data both in terms of the scale of deformations, complexity of the geometry, and the number of fiducial markers used.

The following table shows the variability of our simulation results used to generate our training and testing dataset.

	H&N tumor	Kidney	Aorta
**Max. nodal deformation (mm)**	18.33	25.72	15.74
**Min. nodal deformation (mm)**	0.79	0.45	0.34
**The standard deviation of nodal deformation (mm)**	3.87	6.66	2.14

While it is challenging to find exact reference values for each specific geometry, several papers cite the range of nodal deformations during image guided liver surgery as between 5 to 20mm (Clements et al. 2011 & 2017), as well as aforementioned deformation modeling papers which simulated displacements up to 15mm (Lorente et al. 2017).

Furthermore, we evaluate our framework's performance on the three geometries; H&N tumor, kidney, and aorta aneurysm to add additional geometric complexity. The kidney poses an additional challenge due to its hollow structure, and the aorta due to its hollow structure and thin walls.

With regards to the complexity of fiducial marker displacements used in our framework, the H&N tumor models use just 5 fiducial markers, and the aorta aneurysm uses 10 fiducial markers. Clinically, a minimum of 3 fiducial markers must be implanted. That said, the 10 fiducial markers used in the aorta aneurysm case may exceed what is practical. This is an important clinical consideration, because fiducial markers must be surgically implanted prior to the surgery. For all three cases, we perform a parametric study to select the minimum number of fiducial markers which yields deformation reconstruction error which is below a threshold.

Clements, Logan W., et al. "Deformation correction for image guided liver surgery: An intraoperative fidelity assessment." *Surgery* 162.3 (2017): 537-547.

Clements, Logan W., et al. "Organ surface deformation measurement and analysis in open hepatic surgery: method and preliminary results from 12 clinical cases." *IEEE transactions on biomedical engineering* 58.8 (2011): 2280-2289.

Lorente, Delia, et al. "A framework for modelling the biomechanical behaviour of the human liver during breathing in real time using machine learning." *Expert Systems with Applications* 71 (2017): 342-357.
